# Travel contexts for different forms of multimodality in the new urban mobility landscape: a latent class analysis

**DOI:** 10.1007/s11116-025-10596-8

**Published:** 2025-03-10

**Authors:** Xingxing Fu, Dea van Lierop, Dick Ettema

**Affiliations:** Department of Human Geography and Spatial Planning, Faculty of Geosciences, Utrecht University, 3584 CB Utrecht, The Netherlands

**Keywords:** Multimodality, Modality style, Travel context, New mobility services, Latent class analysis

## Abstract

Multimodality has been recognised as a sustainable way of travel, triggering transport policies to seek solutions that facilitate multimodality. However, although emerging mobility services and transport options in the new urban mobility landscape unlock new possibilities for multimodality, little is known about their roles in different forms of multimodal travel. Therefore, this study investigated the forms of multimodality and their relationship with individual travel contexts considering new trends in the urban mobility sector. In the identification of modality styles, a broader and more detailed set of transport modes was considered; and in the definition of individual travel contexts, a series of factors related to the availability and accessibility of transport options and mobility services were considered. Using latent class analysis, this study identified five modality styles including three forms of multimodality that have not been found in previous research. Distinct forms of public transport (bus, tram, metro, and train) were found to be used in conjunction with other transport modes in different ways, leading to different forms of multimodality. Mopeds and motorcycles, rarely considered in previous research, were found to be the primary travel mode for a small group of people. In addition, weighted multinomial logit regression was used to assess the association between individual travel contexts and modality styles. The results indicate that new mobility services, such as (e-)bike-sharing, have the potential to promote more sustainable forms of multimodality that combine active modes with public transport.

## Introduction

Multimodality, defined as an individual’s use of multiple transport modes within a certain time period (Nobis 2007), has been recognised as an environmentally friendly way to travel (Heinen and Mattioli 2019; Timmer et al. 2023a, b). Existing studies found that multimodal travellers are more likely to travel by public and non-motorised transport modes and less likely to travel by car (Buehler and Hamre 2013; Diana and Mokhtarian 2009a, b). The potential of multimodality to reduce car dependence and relevant environmental problems (such as noise, pollution, and congestion) has therefore motivated research into how multimodal travel behaviour can be encouraged^1^,^2^ Meanwhile, the introduction of new transport modes and mobility services (such as shared mobility, ride-hailing and Mobility-as-a-Service) into urban mobility systems presents opportunities for new forms of multimodal travel (Buehler and Hamre 2016; Klinger 2017; Rafiq and McNally 2022). Therefore, understanding different forms of multimodality in the new urban mobility landscape can provide insights for transport policies and planning aimed at facilitating multimodality.

In transport research, the term “multimodal” refers to the combination and coordination of various transport modes (EC, 2014) and is used to describe both the supply and demand sides of transport. On the supply side, “multimodal” is used to characterise transport facilities that offer passengers multiple transport options, mobility services designed to enable seamless journeys across a multitude of transport modes, as well as relevant mobility policies and business models (Kuhnimhof et al. 2006). On the demand side, “multimodal” is used to characterise individuals’ way of travelling as well as associated personal attitudes, describing passengers’ demand for various transport modes (Diana and Mokhtarian 2009a, b; Mattioli and Heinen 2020). In this study, we focus on the demand side of multimodality, and multimodality in the following text refers to multimodal travel behaviour. The current research on multimodality can be divided into two types, one focusing on explaining the *degree* of multimodality, and the other focusing on explaining the *forms* of multimodality. Research on the *degree* of multimodality uses continuous indicators to measure the diversity and evenness of transport modes used by individuals (Diana and Pirra 2016; Fu et al. 2023; Heinen and Chatterjee 2015), analysing whether and to what extent travellers are multimodal. Research on the *forms* of multimodality classifies individuals based on the combinations and ratios of transport modes they use (Diana and Mokhtarian 2009a, b; Olafsson et al. 2016). These studies attempt to reveal individuals’ modality styles (i.e. travel patterns of using transport modes) according to the classification (Shamshiripour et al. 2020; Vij et al. 2013), which often encompass different forms of multimodality as well as monomodal travel patterns. With the emergence of new transport modes and services, modality styles are diversifying, and multimodality is becoming increasingly possible (Buehler and Hamre 2016; Klinger 2017; Rafiq and McNally 2022).

However, to date, the majority of studies identifying modality styles have limited the mode set to the most common modes: walking, cycling, private car, and public transport (Kroesen 2014; Lu et al. 2021). Few studies made the distinction between different forms of public transport such as bus, tram, metro and train, which are grouped together as a general public transport mode instead (De Haas et al. 2018; Krueger et al. 2018). Consequently, the identified modality styles are often not very multimodal, with one mode dominating (Lee et al. 2020; Molin et al. 2016), and often differ only in the proportion of modes (with the same modal combination) (Ton et al. 2020). Therefore, the complexity of modality styles, especially diverse multimodal patterns, is underexplored. Capturing this complexity is important to understand how individuals organise their daily travel by combining specific transport options. For instance, the role of local bus in individuals’ daily travel is not well understood if it is grouped into the broader category of public transport.

Hence, to capture the distinction and variety of modality styles, a broader and more detailed set of transport options needs to be considered. First, besides the most common modes, other travel modes such as the use of mopeds, motorcycles and taxis may also matter in the identification of modality styles. For example, according to the Dutch National Travel Survey (ODiN), in 2021, 8.73% of passenger kilometres were travelled by transport modes other than walking, cycling, car and public transport, and in several provinces, this proportion even exceeded 10%^3^. Also, with the development of an array of new mobility services including on-demand mobility and shared mobility, taxi, ride-hailing, shared e-scooter and shared e-moped become more frequently used (Janke et al. 2021; Lee et al. 2020). Second, since different forms of public transport including bus, tram, metro and train often serve different destinations and different travel distances (Molin et al. 2016), in most regions, it is logical to treat them as different modes when analysing multimodality (Lavery et al. 2013). Likewise, car use may vary in the role of drivers and passengers. Clearly, using a broader and more detailed set of transport options allows one to better grasp the differences in the forms of multimodality, which may also help transport authorities and regional governments better understand different segments of travellers (Jacques et al. 2013).

In addition to identifying modality styles, to gain a deeper understanding of the *forms* of multimodality, it is also important to examine which personal and contextual factors influence an individual’s modality style. Studies have already investigated the associations between modality styles and socioeconomic characteristics such as income and education, psychological characteristics such as travel-related attitudes and social norms, as well as spatial characteristics including neighbourhood types and urban forms (Krueger et al. 2018; Lee et al. 2020; Molin et al. 2016; Ton et al. 2020). Since transport policies and planning have limited impact on the aforementioned factors, more insight is needed into the influence of factors describing an individual’s travel context, such as the availability of transport options, accessibility of transport facilities, quality of transport services, and application of new mobility services.

However, these travel contextual factors have been rarely addressed in research on the *forms* of multimodality. A variety of travel contextual factors including vehicle and licence ownership (An et al. 2021; Heinen and Chatterjee 2015), access to transport facilities (Buehler and Hamre 2016; Heinen and Chatterjee 2015), and the use of mobility services (Astroza et al. 2017) have been assessed for their effects on the *degree* of multimodality. However, apart from vehicle and licence ownership, the effects of other contextual factors on the *forms* of multimodality have hardly been assessed. Investigating the effects of travel contexts on the *forms* of multimodality is needed for several reasons. First, there is debate about whether individuals are multimodal by choice or by necessity because findings on the relationship between travel contexts and the *degree* of multimodality are mixed (Buehler and Hamre 2015; Kuhnimhof et al. 2006). Different travel contexts may influence not only whether and to what extent an individual is multimodal, but also the combination of transport modes they use, and different mode combinations may stem from different motivations (Fu et al. 2024). For example, those who have no access to a car and limited access to public transport may have to transfer between different public transport modes in order to reach their destinations, thus using a combination of walking and different forms of public transport by necessity (Buehler and Hamre 2015); those who enjoy various travel options have the privilege to choose the optimal mode for each given trip, thus using a combination of transport modes like active modes (for short distances and in good weather) and car (for long distances and in bad weather) by choice (Faber et al. 2022). Therefore, examining the relationship between travel contexts and different forms of multimodality may add empirical evidence for this debate. Second, new mobility services, such as shared mobility and online trip planning, provide flexible options and more information to enable multimodal travel, but their effects on both the *degree* and *forms* of multimodality have not yet been assessed. Given the increasingly significant role of new mobility services in urban mobility systems, it is worthwhile to investigate their relationship with multimodality.

From the above, we conclude that while the evolution of mobility has redefined urban residents’ transport option sets and unlocked new possibilities for various forms of multimodality, research on the forms of multimodality is still limited to a narrow set of transport modes and has used a limited account of travel contexts. This study aims to investigate the forms of multimodality and their relationship with travel contexts in a more comprehensive way, accounting for a broader and more detailed set of transport modes in the identification of modality styles and a series of travel contextual factors in the definition of an individual’s travel context. Based on survey data from two large Dutch cities, the study uses bias-adjusted three-step latent class analysis to identify modality styles based on respondents’ use of ten transport modes (including distinct types of public transport, car driver and car passenger, moped/motorcycle, and taxi/ride-hailing) and examine the associations between travel contexts and the probability of belonging to different modality styles.

In the next section, we provide an overview of the factors related to modality styles. Sect. "Data and methods" presents the conceptual framework of this study, and describes the data and methods used for the analysis. Section "Results" presents the results of the analysis. Sect. "Discussion" discusses the key results, policy implications, limitations, and possible future research directions. Sect. "Conclusion" summarises the main research findings.

## Factors related to modality styles

*Socio-demographics* have been investigated for their associations with modality styles in most previous studies (Krueger et al. 2018; Lee et al. 2020; Molin et al. 2016; Ton et al. 2020). Modality styles are found to be related to personal attributes including age, gender, education level and occupational status, and household attributes including household income and household composition (Kroesen 2014; Kroesen and Van Cranenburgh 2016; Lee et al. 2020; Molin et al. 2016; Ton et al. 2020). Since the modality styles identified in different studies vary, it is difficult to compare their results. Still, consistent results were found in a few studies, with young, highly educated people being more likely to belong to public transport-based multimodal patterns compared to other modality styles (Molin et al. 2016; Ton et al. 2020).

*Spatial factors* at both city and neighbourhood levels have been investigated for their associations with modality styles in several studies. Residential city size, neighbourhood location and density were found to be related to modality styles (Lee et al. 2020; Molin et al. 2016). Specifically, people living in highly urbanised areas are more likely to belong to public transport-based multimodal patterns or cycling-based monomodal patterns compared to car-based modality styles (both monomodal and multimodal) and cycling-based multimodal patterns (Olafsson et al. 2016; Ton et al. 2020), while those living in suburban areas are more likely to belong to car-based modality styles (both monomodal and multimodal) compared to other modality styles (Lee et al. 2020). However, spatial factors can be highly correlated with the availability and accessibility of transport facilities and services, and these objective travel contextual factors were barely considered in previous studies.

*Objective travel contextual factors*, including vehicle ownership, accessibility to public transport and the availability of slow mobility facilities have been found to induce or constrain the use of a certain transport mode (Handy et al. 2005; Ma et al. 2021; Vovsha et al. 2013). Whereas in previous studies on modality styles, only vehicle ownership and licence ownership were commonly tested as predictors. Other travel contextual factors, such as access to public transport and other transport facilities in residence may be related to modality styles while have hardly been accounted for. For example, public transport is more likely to be jointly used with other transport modes due to the ‘first/last mile problem’ (Lu et al. 2022), so living close to a subway station was found to increase both the use of subway and cycling (Ma et al. 2021). Thus, better access to public transport may be related to modality styles with a combination of public transport and active modes. Likewise, other objective travel contextual factors, such as the availability of walking and cycling facilities may also be related to certain modality styles.

In addition, a specific set of objective travel contextual factors, *new mobility services*, such as shared mobility and on-demand mobility, are booming in the urban transport sector. These services are changing the way of accessing and paying for certain forms of mobility, and thus modifying people’s travel option sets (Castellanos et al. 2021; Chen et al. 2022; Rafiq and McNally 2022). Therefore, new mobility services may offer the possibility of various forms of multimodality. However, mobility services considered in previous studies are limited to subscriptions to public transport (Lee et al. 2020; Ton et al. 2020). It is desirable to understand how the use of new mobility services is related to modality styles, which may uncover the potential of new mobility services to facilitate more sustainable forms of multimodality (Zhou et al. 2022).

*Subjective travel attributes* may also be related to modality styles. Previous studies have found that attitudes toward travel, mode perceptions and preferences, and social norms are related to modality styles (Krueger et al. 2018; Lee et al. 2020; Molin et al. 2016; Ton et al. 2020). Molin et al. (2016) found that those having negative attitudes toward public transport are more likely to belong to car-based modality styles (both monomodal and multimodal) compared to cycling-based multimodal patterns and public transport-based multimodal patterns, and Ton et al. (2020) found that having a positive attitude toward cars is strongly associated with being a monomodal car user.

## Data and methods

### Model conceptualization

**Fig. 1:**
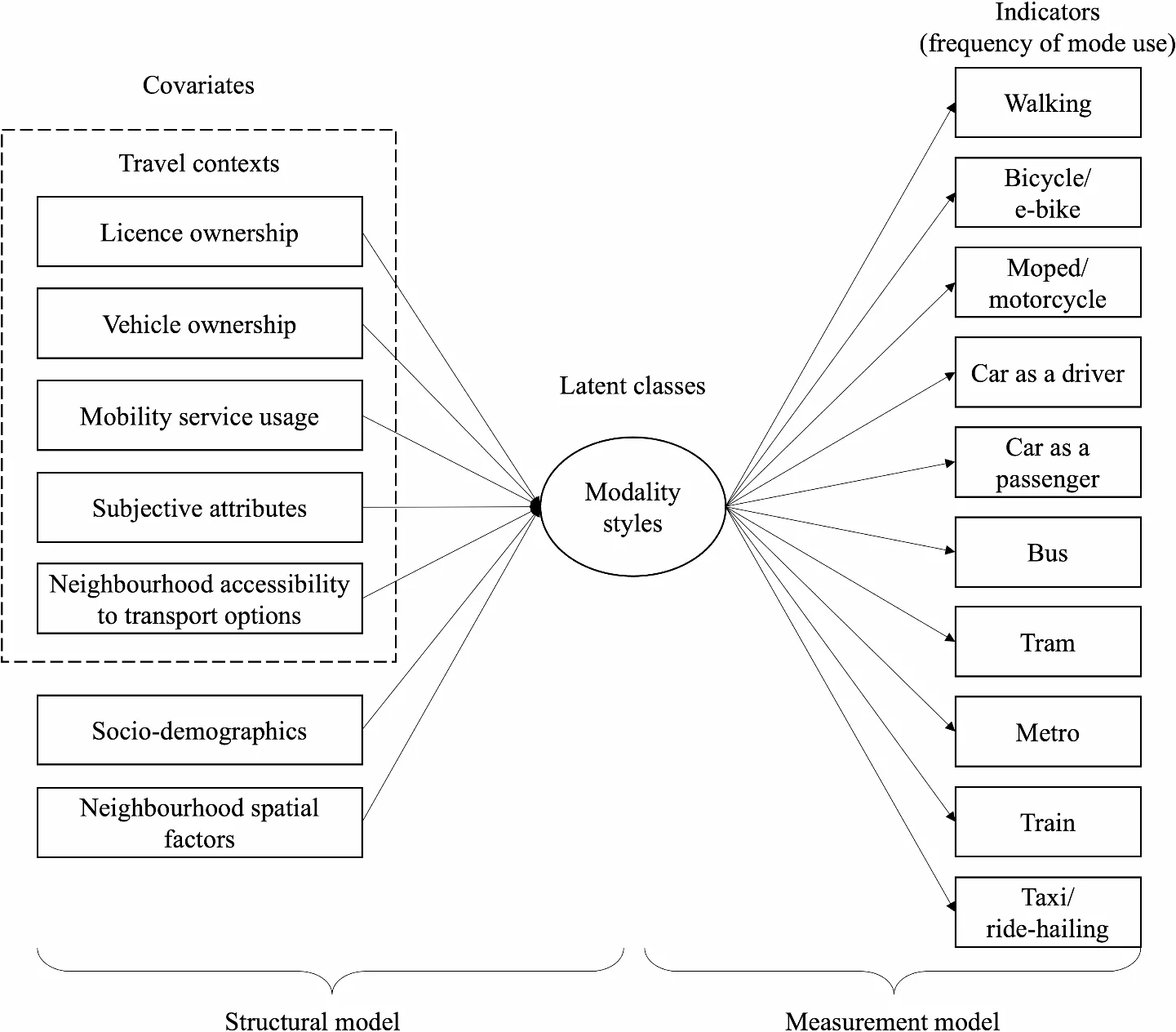
Conceptual model

Latent class analysis is used to investigate the relationship between individuals’ travel contexts and modality styles (Fig. 1). Modality styles are classified according to a group of people’s frequency of using different transport modes. A broad set of transport modes are considered, including walking, bicycle/e-bike, moped/motorcycle, taxi/ride-hailing, and distinctions are made between bus, tram, metro and train, and car driver and car passenger.

Based on this classification of modality styles, each individual is assigned a class (modality style) to which they are most likely to belong. Then the associations between individuals’ travel contexts and modality styles are assessed. Five aspects of travel contextual factors, including *licence ownership*, *vehicle ownership*, *mobility service usage*, *subjective attributes*, and *neighbourhood accessibility to transport options* are used to define an individual’s travel context. Since socio-demographics and spatial factors may have associations with both travel contexts (e.g., people get a driving licence or own a personal car until they are of a certain age) and modality styles, we control for socio-demographics and neighbourhood spatial factors in the model to avoid possible confounding effects.

### Data and measures

This study uses data from a survey conducted in the summer of 2021 in Rotterdam and Utrecht, two major cities in the Netherlands. Rotterdam is the second largest city in the Netherlands, with a population of 651,631 in 2021. Rotterdam has a local public transport system with bus, tram and metro. Rotterdam is one of the most car-dependent cities in the Netherlands (with a mode share of 38%^4^). Utrecht is the fourth largest city in the Netherlands, with a population of 359,370 in 2021. Utrecht has a local public transport system with bus and tram. In Utrecht, journeys by bicycle are more than any other mode of transport (with a mode share of 51%^5^).

The survey is part of a project on the theme of transport equity, which aims to quantitatively investigate the extent, causes and effects of urban residents’ access to transport in the Netherlands. Participants were recruited by two agencies (Labyrinth and PanelClix), targeting the age group of 18–70 years old. 1203 participants were recruited in total, and each participant was asked to fill in an online questionnaire. Before the survey was launched online, the individual questions were pretested in face-to-face sessions to ensure that the questions were relevant and understandable. The survey collected information on individuals’ travel behaviour, travel attributes and travel experiences. In addition, we extracted detailed data on land use, transport facilities and accessibility as neighbourhood-level variables by linking individuals’ residential PC4 (4-digit postal code) addresses to publicly available data. We selected cases that completed the survey and reported their PC4 was within Utrecht or Rotterdam as our research sample. The sample size of the current analysis is 1016, among which 496 are from Rotterdam and 520 are from Utrecht. Variables used in the analysis include frequencies of mode use as indicators of modality styles, and socio-demographics, neighbourhood spatial factors, and travel contextual factors as covariates of modality styles (see Table 8 in Appendix for an overview of the variables and their data sources).

**Fig. 2:**
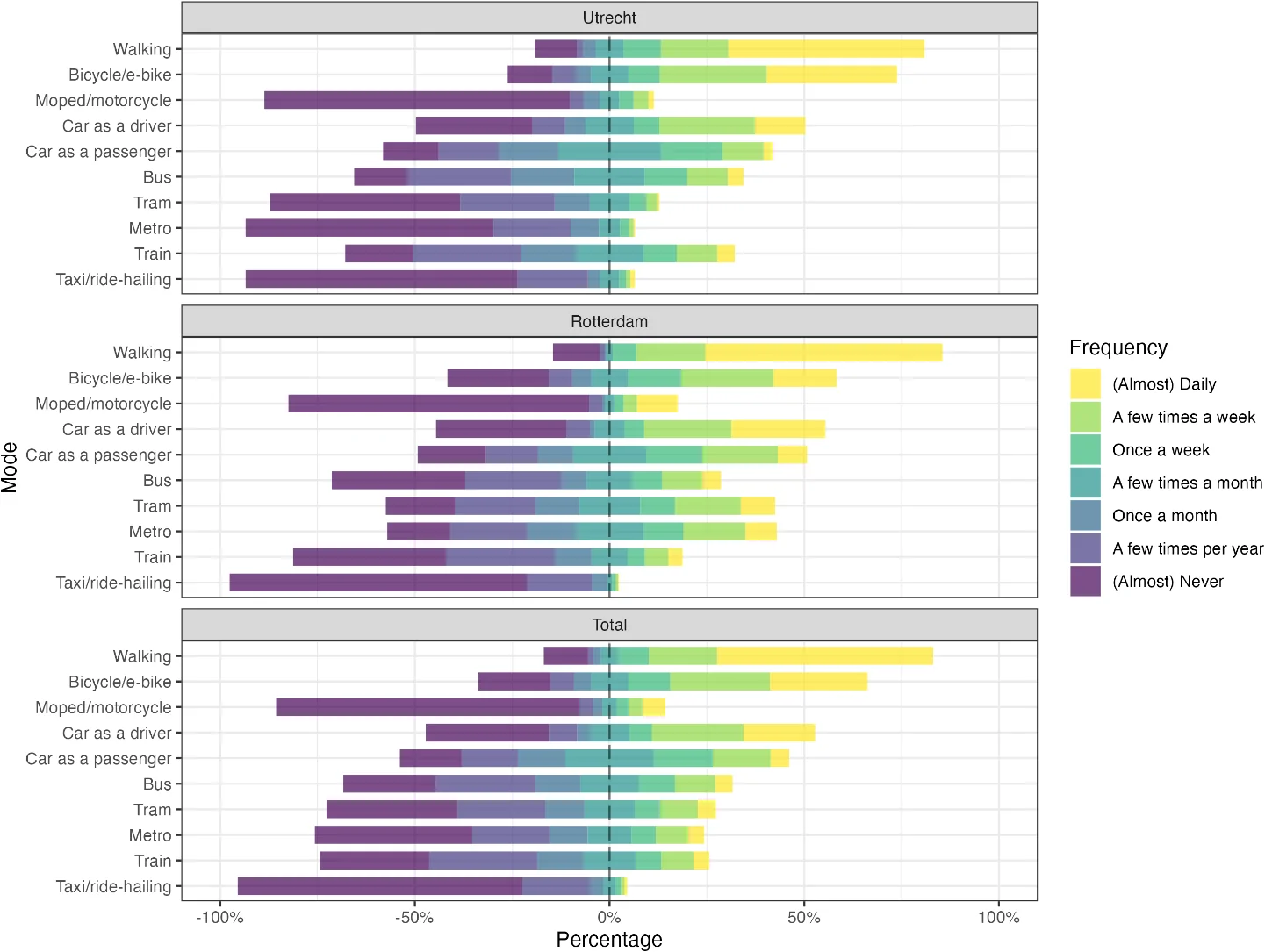
Distribution of mode use frequency

Frequencies of mode use were derived from a section of the questionnaire that recorded how often an individual used various modes of transport. We chose ten modes among thirteen because almost 90% of respondents reported that they never used e-scooters, cargo-bikes or regiotaxis (a kind of demand-responsive public transport from door to door). The ten modes are: *walking*, *bicycle/e-bike*, *moped/motorcycle*, *car as a driver*, *car as a passenger*, *bus*, *tram*, *metro*, *train* and *taxi/ride-hailing*. Answers to these questions are based on a seven-point scale from 1 ‘almost never’ to 7 ‘almost daily’, so we used them as ordinal variables in the analysis. Figure 2 shows the distribution of mode use frequency for the two city subsamples and the entire sample. Walking and cycling are the most frequently used modes for both subsamples, followed by car (both as drivers and passengers) and different forms of public transport. Utrecht subsample has a higher percentage of people who frequently use bicycle/e-bike, bus and train, and Rotterdam subsample has a higher percentage of people who frequently use moped/motorcycle, car (both as drivers and passengers), tram and metro.

Socio-demographics include *age*, *gender*, *working status*, *education level*, *living with children*, and *household income* (Table 1). It should be noted that our sample contains seven non-binary/genderfluid individuals, and we combined them with females to create a non-male category in the analysis, because by comparison, we found that their mode use frequency was closer to females’. Two neighbourhood spatial factors, *address density* and *greenery*, were used as comprehensive proxies of land use and environment (Table 1).*Addressdensity* is from CBS^6^, which calculated the average number of addresses per square kilometre within a PC4. *Greenery* is from the RIVM^7^, which calculated the average number of trees, shrubs and plants on a surface of 10 by 10 m within a PC4.

**Table 1:** Description of socio-demographics, neighbourhood spatial factors and travel contexts

	Utrecht		Rotterdam		Independence test χ^2^/F (*p*-value)	Sample total	
	N	Percent %	N	Percent %		N	Percent %
*Socio-demographics*							
Age class					9.65 (0.086)		
< = 20	42	8.08	20	4.03		62	6.10
21–30	176	33.85	155	31.25		331	32.58
31–40	120	23.08	124	25.00		244	24.02
41–50	63	12.12	73	14.72		136	13.39
51–60	67	12.88	73	14.72		140	13.78
> = 61	52	10.00	51	10.28		103	10.14
Gender					**6.69 (0.035)**		
Male	201	38.65	229	46.17		430	42.32
Female	314	60.38	265	53.43		579	56.99
Non-binary/genderfluid	5	0.96	2	0.40		7	0.69
Working status					**15.54 (0.001)**		
Full-time	167	32.12	216	43.55		383	37.70
Part-time	190	36.54	149	30.04		339	33.37
Student	81	15.58	56	11.29		137	13.48
No job/retired	82	15.77	75	15.12		157	15.45
Education level					**38.20 (0.001)**		
Low education	57	10.96	83	16.73		140	13.78
Medium education	139	26.73	200	40.32		339	33.37
High education	324	62.31	213	42.94		537	52.85
Living with children					0.39 (0.532)		
Yes	109	20.96	112	22.58		221	21.75
No	411	79.04	384	77.42		795	78.25
Household income					1.87 (0.760)		
Less than €980	74	14.23	74	14.92		148	14.57
Between €980 and €1870	127	24.42	116	23.39		243	23.92
Between €1870 and €2680	119	22.88	111	22.38		230	22.64
Between €2680 and €3800	100	19.23	109	21.98		209	20.57
More than €3800	21	4.04	15	3.02		36	3.54
Don’t know/don’t want to say	79	15.19	71	14.31		150	14.76
*Neighbourhood spatial factors*							
Address density (/km^2^) (mean)	3218.63		4278.79		**145.39 (0.000)**	3736.19	
Greenery (mean)	47.69		41.65		**96.32 (0.000)**	44.75	
*Travel contexts*							
Holding driver’s licence					0.00 (0.950)		
Yes	371	71.35	353	71.17		724	71.26
No	149	28.65	143	28.83		292	28.74
Car ownership					**8.65 (0.013)**		
Personal car	192	36.92	228	45.97		420	41.34
Household car	57	10.96	49	9.88		106	10.43
No car	271	52.12	219	44.15		490	48.23
Bicycle/e-bike ownership					**28.26 (0.000)**		
Yes	458	88.08	373	75.20		831	81.79
No	62	11.92	123	24.80		185	18.21
Public transport subscription					1.33 (0.248)		
Yes	207	39.81	180	36.29		387	38.09
No	313	60.19	316	63.71		629	61.91
Car-sharing membership					**5.91 (0.015)**		
Yes	31	5.96	14	2.82		45	4.43
No	489	94.04	482	97.18		971	95.57
(E-)Bike-sharing membership					**12.04 (0.000)**		
Yes	60	11.54	27	5.44		87	8.56
No	460	88.46	469	94.56		929	91.44
Planning trips by smartphone					2.06 (0.151)		
Yes	274	52.69	239	48.19		513	50.49
No	246	47.31	257	51.81		503	49.51
Feeling the ease of driving					**4.25 (0.039)**		
Yes	214	41.15	236	47.58		450	44.29
No	306	58.85	260	52.42		566	55.71
Perceiving good PT quality					2.76 (0.096)		
Yes	285	54.81	246	49.60		531	52.26
No	235	45.19	250	50.40		485	47.74
Walking way density (/km^2^) (mean)	11.60		8.95		**93.30 (0.000)**	10.31	
Cycling way density (/km^2^) (mean)	6.86		5.80		**68.98 (0.000)**	6.34	
Bus frequency density (/km^2^) (mean)	105.02		86.47		**30.33 (0.000)**	95.96	
Tram/metro frequency density (/km^2^) (mean)	2.39		202.06		**324.70 (0.000)**	99.87	
Distance to the nearest main road access ramp (km) (mean)	1.99		2.45		**76.58 (0.000)**	2.21	
Distance to the nearest interchange train station (km) (mean)	4.02		5.13		**60.13 (0.000)**	4.56	
Distance to the nearest train station (km) (mean)	1.77		2.55		**79.60 (0.000)**	2.15	
Total	520	100.00	496	100.00		1016	100.00

Independence test results with a significance level of p<0.05 are marked in bold

Individual-level travel contextual factors include *vehicle ownership*, *licence ownership*, *mobility service usage* and *subjective attributes* (Table 1). Considering there might be competition among family members sharing one car, we distinguished between personal cars and household cars: those who reported having a personal car were classified in the *personal car* category; those who reported having household cars but no personal car were classified in the *household car* category; and those who reported having neither a personal car nor household car were classified in the *no car* category. Moped/motorcycle ownership was excluded based on the data description because it has a one-to-one relationship with the use of moped/motorcycle. Since only 10 participants reported using moped-sharing, we also excluded moped-sharing membership to avoid estimation bias.

Subjective attributes were processed by exploratory factor analysis. A battery of nine items about individuals’ feelings about driving and public transport were used to measure individuals’ perceptions of transport modes (Table 2). Individuals were asked to report to what extent they agree with each item on a five-point Likert scale ranging from (1) completely disagree to (5) completely agree. One single EFA was used to reduce the dimension of the nine items. Before the EFA, we did a reliability test on the scale, and Cronbach’s Alpha was 0.727, suggesting the nine items have relatively high internal consistency. We used Principal Axis Factoring extraction and Promax rotation for the EFA, and we decided the number of extracted factors by eigenvalue greater than 1. Two factors were generated, with one mainly explaining items related to driving and the other mainly explaining items related to public transport. We named the two factors as *feeling the ease of driving* and *perceiving good public transport quality* and produced factor scores by regression method. The produced scores have a mean of 0 and a variance equal to the squared multiple correlation between the estimated factor scores and the true factor values. We further transformed the factors into binary variables for use as covariates in the structural model. For each factor, if an individual scored above the average (that is, with a positive value), the person was defined as being in the “Yes” category of the corresponding variable; if the score was zero or negative, then the individual was defined as being in the “No” category of the corresponding variable.

**Table 2:** Factor analysis of subjective attributes

Item	Factor 1	Factor 2
	Feeling the ease of driving	Perceiving good public transport quality
I feel comfortable driving a car	**0.920**	0.042
I have a lot of experience driving a car	**0.826**	0.041
I find it hard to drive under difficult conditions	**− 0.586**	− 0.026
I prefer not to drive	**− 0.762**	− 0.001
Public transport is affordable to me	0.009	**0.544**
Public transport is easy to understand how to use	0.026	**0.692**
Public transport is accessible to people with reduced mobility	0.076	**0.508**
Public transport is available at times that are useful to me	− 0.004	**0.750**
Public transport reaches destinations or activities that are important to me	0.010	**0.716**
Extraction Sums of Squared Loadings (% of Variance)	27.496	23.237
Kaiser–Meyer–Olkin Measure of Sampling Adequacy: 0.786		
Bartlett's Test of Sphericity Significance Level: 0.000		

Extraction Method: Principal Axis Factoring

Rotation Method: Promax with Kaiser Normalization

Bold indicates that the factor of the corresponding column mainly explains the item

Neighbourhood accessibility to transport options was mainly from two sources. We used walking road and cycling road layers from Open Street Map to calculate the average length of walking way and cycling way per square meter in a PC4 as the variables *walking way density* and *cycling way density*. We used the schedule of buses from GTFS^8^ data to calculate the number of bus lines passing through all bus stops in a PC4 per day, and divided the total number by the area of the PC4 as the variable *bus frequency density*. We used the schedule of trams and metros from GTFS data to calculate the number of tram and metro lines passing through all tram and metro stations in a PC4 per day, and divided the total number by the area of the PC4 as the variable *tram/metro frequency density*. We merged the frequency densities of tram and metro because they are highly correlated and may cause multicollinearity problems in the models. *Distance to the nearest main road access ramp*, *distance to the nearest train station* and *distance to the nearest interchange train station* were all from CBS^9^, and these data were calculated by the average road network distance of all residents in a PC4 to the nearest facilities.

We conducted independence tests to compare the variables of the two cities’ subsamples. Chi-squared tests were used for comparing the distribution of categorical variables and one-way ANOVA tests were used for comparing continuous variables. The tests show that the two cities’ subsamples differed significantly in several variables of socio-demographics, built environment, vehicle ownership and objective access to transport options (Table 1). In terms of socio-demographics, the Utrecht subsample has a higher proportion of females, people with part-time jobs or students, and people with high education. The Rotterdam subsample has a higher average address density than Utrecht, but lower average greenery. The Rotterdam subsample has a higher proportion of people having a personal car, and the Utrecht subsample has a higher proportion of people having a personal (e-)bike or being (e-)bike-sharing members. The Utrecht subsample has higher average access to walking and cycling facilities, bus lines, main road access ramps and train stations. The Rotterdam subsample has higher average access to tram/metro lines, as there are more tram lines as well as a metro system which is not available in Utrecht. Given the significant city differences in travel contexts, we add the binary variable *city* in the structural model as a covariate to control for the unaccounted-for city differences.

### Model specification: latent class analysis

Latent class analysis (LCA) is a statistical procedure identifying several classes within a latent variable measured by a set of observed indicators (Bakk and Kuha 2018; Goodman 1974; McCutcheon 1987). LCA has been widely used to identify modality styles by accounting for the associations between the use of different transport modes (Lee et al. 2020; Lu et al. 2022; Molin et al. 2016). To further analyse the predictors or outward characteristics of latent classes, the basic LCA can be extended by using observed covariates to predict latent class membership (Vermunt 2010). Compared to conventional clustering techniques, latent class analysis has several advantages when fitting the conceptual model of this study. First, the model-based solution allows for the identification of underlying patterns in observed data based on statistical information, rather than relying on predefined factors or clusters, thus enabling a more nuanced understanding of the underlying structure of a series of dispersed objects. Second, latent class analysis provides several statistical tests to assess model fit, so we can use formal criteria to compare the performance of models with different numbers of classes and decide the appropriate number, which is more flexible and rigorous. Third, latent class analysis assigns individuals to each class by certain probabilities instead of deterministically restricting one to a single class, so we can consider the weights of individuals in each class and account for misclassification errors when relating latent classes to external variables, which is less biased. Therefore, in this study, we use LCA to measure modality styles by mode use frequency and predict modality styles with travel contexts (Fig. 1).

Consider the vector of indicators ${{\boldsymbol{Y}}}_{{\boldsymbol{i}}}= \left({Y}_{i1}, {Y}_{i2},\dots, {Y}_{ik}\right)$, where ${Y}_{ik}$ denotes individual *i*’s frequency of using transport mode *k*. In our analysis, *K=10* is the number of transport modes considered and *N=1016* is the number of individuals in the sample. LCA assumes there is a categorical latent variable *X* that explains associations between indicators, and individual *i* has a certain probability of belonging to each of the *T* latent classes depending on ${{\boldsymbol{Y}}}_{{\boldsymbol{i}}}$. The LCA model can then be written as:

1 $$p\left({{\boldsymbol{Y}}}_{{\boldsymbol{i}}}\right)=\sum_{t=1}^{T}p\left(X=t\right)p\left({{\boldsymbol{Y}}}_{{\boldsymbol{i}}}\mid X=t\right)$$

where $p\left(X=t\right)$ is the (unconditional) probability that an individual belongs to latent class *t*, and $p\left({{\boldsymbol{Y}}}_{{\boldsymbol{i}}}\mid X=t\right)$ is the class-specific probability of an individual’s responses to the indicators given the latent class *t*. Making the ‘local dependence’ assumption that the *K* observed indicators are independent within the latent classes, leading to:

2 $$p\left({{\boldsymbol{Y}}}_{{\boldsymbol{i}}}\right)=\sum_{t=1}^{T}p\left(X=t\right)\prod_{k=1}^{K}p\left({Y}_{ik}\mid X=t\right)$$

This is referred to as the *basic LCA model*, where covariates are not taken into account. As the identification of latent classes focuses on explaining the associations between the observed indicators, we use the basic LCA model to determine the appropriate number of classes (Bakk and Kuha 2021). The number of classes *T* is determined by comparing the goodness-of-fit of models with different values of *T* using a series of criteria including log-likelihood, Akaike information criterion, Bayesian information criterion, and entropy (values closer to 1 indicate better separation between classes) (Bakk and Kuha 2021; Magidson 1981; Vermunt 2010).

When considering covariates of the latent class variable *X*, denoted here by the vector ${{\boldsymbol{Z}}}_{{\boldsymbol{p}}{\boldsymbol{i}}}$, then the LCA model can be extended to:

3 $$p\left({{\boldsymbol{Y}}}_{{\boldsymbol{i}}}\mid {{\boldsymbol{Z}}}_{{\boldsymbol{p}}{\boldsymbol{i}}} \right)=\sum_{t=1}^{T}\left[p\left(X=t\mid {{\boldsymbol{Z}}}_{{\boldsymbol{p}}{\boldsymbol{i}}}\right)\prod_{k=1}^{K}p\left({Y}_{ik}\mid X=t\right)\right]$$

by replacing the unconditional probability $p\left(X=t\right)$ in the basic LCA model with $p\left(X=t\mid {{\boldsymbol{Z}}}_{{\boldsymbol{p}}{\boldsymbol{i}}}\right)$, which is the probability of belonging to a specific class *t* given an individual’s covariates ${{\boldsymbol{Z}}}_{{\boldsymbol{p}}{\boldsymbol{i}}}$. In this extended LCA model, the basic component measuring latent classes by indicators is defined as the measurement model, and the extended component predicting the latent class membership with covariates is defined as the structural model.

### Model estimation: bias-adjusted three step approach

Most of LCA-based studies on modality styles used the one-step approach (Lee et al. 2020; Molin et al. 2016; Ton et al. 2022), simultaneously estimating the measurement model and the structural model Eq. (3) to obtain maximum likelihood (ML) estimates for all parameters (Bakk and Kuha 2018; Dayton and Macready 1988). Although the approach provides asymptotically unbiased, efficient or consistent estimates of the relationships among all variables (Bakk and Kuha 2021; Bolck et al. 2004; Vermunt 2010), there are certain defects. First, the measurement and structural components of the model influence each other, which easily leads to unstable model results and distortion of model interpretation (Bakk and Kuha 2018, 2021). Second, the whole model is always refitted even when only one part of the model is changed, such as removing or adding one covariate, so the approach is not suitable for comparisons between models with different covariates (Bakk and Kuha 2021; Bolck et al. 2004). However, few studies checked possible distortions, and some even compared models with different sets of covariates and determined the final model by comparing goodness-of-fit (Lee et al. 2022; Ton et al. 2022, 2020).

To prevent distortion of model interpretation, this study uses a three-step approach to estimate different components of LCA step by step. The three-step approach of LCA involves:

1. Estimating the measurement model by fitting the basic LCA model Eq. (2) with the determined number of latent classes and selecting the optimal number of classes based on goodness-of-fit.
2. Computing posterior class membership probabilities given the respondent’s response vector ${{\boldsymbol{Y}}}_{{\boldsymbol{i}}}$ and the parameters estimated in Step 1. Assigning individuals to latent classes ${t}_{i}$ which have the highest posterior membership probability.
3. Estimating the structural model with the assigned classes ${t}_{i}$ and covariates ${{\boldsymbol{Z}}}_{{\boldsymbol{p}}{\boldsymbol{i}}}$ using multinomial logit regression.

Considering the possible classification errors in Step 2, we use ML bias-adjusted method to improve the three-step approach (Bakk et al. 2013; Bolck et al. 2004; Vermunt 2010): each individual is assigned to all latent classes with weights equal to posterior probabilities belonging to the corresponding latent classes (Bakk et al. 2013; Vermunt 2010). Specifically, the model estimates the following equation:

4 $$p\left({t}_{i}\mid {{\boldsymbol{Z}}}_{{\boldsymbol{p}}{\boldsymbol{i}}} \right)=\sum_{t=1}^{T}p\left(X=t\mid {{\boldsymbol{Z}}}_{{\boldsymbol{p}}{\boldsymbol{i}}}\right)p\left({t}_{i}\mid X=t\right)$$

in which $p\left(X=t\mid {{\boldsymbol{Z}}}_{{\boldsymbol{p}}{\boldsymbol{i}}}\right)$ is freely estimated and $p\left({t}_{i}\mid X=t\right)$ is fixed as the weights. The quantities of the weights are the posterior class membership probabilities computed from step 2, and the dataset is thus expanded to contain *T* records per individual with the weights (Vermunt and Magidson 2013). Robust standard errors are used in the estimation of the weighted multinomial logit regression in Step 3 (Bakk et al. 2014). The professional latent class software LatentGOLD 6.0 is used for the analysis.

## Results

### Selection of the number of classes

Table 3 provides the goodness-of-fit results of basic models with 1–10 latent classes. The goal of comparison is to find the most parsimonious model that adequately explains distinct latent characteristics with the smallest number of classes. There were increases in Log Likelihood and decreases in AIC and SA-BIC for all models ranging from 1-class to 10-class, and decreases in BIC for models ranging from 1-class to 7-class. However, differences in Log Likelihood, AIC, BIC and SA-BIC were minimal after the 5-class model. Although the VLMR *p*-value shows that the 6-class model was significantly better fitted than the 5-class model, the entropy score indicated that the 5-class model delineated classes better than the 6-class model. Therefore, parsimony and interpretability supported the selection of the 5-class model.

**Table 3:** Goodness-of-fit indices for basic LCA models

Model	Npar	df	Log likelihood	AIC	BIC	SA-BIC	Entropy	VLMR(*p*)
1 class	60	956	− 15771.425	31662.851	31958.268	31767.703	1.000	
2 class	71	945	− 15274.559	30691.119	31040.696	30815.194	0.799	0.000
3 class	82	934	− 15081.937	30327.875	30731.612	30471.173	0.821	0.006
4 class	93	923	− 14921.342	30028.683	30486.581	30191.205	0.798	0.000
**5 class**	**104**	**912**	**− 14834.846**	**29877.692**	**30389.749**	**30059.436**	**0.782**	**0.000**
6 class	115	901	− 14771.921	29773.842	30340.060	29974.810	0.768	0.037
7 class	126	890	− 14698.166	29648.333	30268.710	29868.523	0.785	0.009
8 class	137	879	− 14672.355	29618.709	30293.246	29825.322	0.789	0.036
9 class	148	868	− 14618.265	29532.530	30261.227	29802.112	0.796	0.000
10 class	159	857	− 14588.486	29494.979	30277.836	29774.511	0.810	0.077

Npar = Number of parameters; df = Degree of freedom; AIC = Akaike Information Criteria; BIC = Bayesian Information Criteria

SA-BIC = Sample-size adjusted Bayesian Information Criteria; VLMR(*p*) = Vuong-Lo-Mendell-Rubin likelihood ratio test *p-value*

Bold indicates the selected model

### Characteristics of the five modality styles

Modality styles were defined according to the distribution (%) of frequency of using different transport modes within each latent class (Table 4). The five identified modality styles are termed (1) *Multimodal*, (2) *Drive mostly*, (3) *Active* + *Train*, (4) *PT multimodal*, and (5) *Moped/motorcycle multimodal*. Table 5 describes the characteristics of each modality style. F-values and chi-square values were statistically significant for all characteristics we included in the analysis, so modality styles differ in socio-demographics, neighbourhood spatial factors, and travel contextual factors.

**Table 4:** Profiles of modality styles

	Class 1 (n = 381)	Class 2 (n = 308)	Class 3 (n = 198)	Class 4 (n = 91)	Class 5 (n = 38)	Overall (n = 1016)
*Walking*						
(Almost) daily	**48.01%**	**50.04%**	**66.14%**	**64.26%**	**94.64%**	**55.51%**
A few times a week	17.72%	18.00%	18.34%	18.50%	4.98%	17.52%
Once a week	8.76%	8.67%	6.81%	7.14%	0.35%	7.87%
A few times a month	5.31%	5.12%	3.10%	3.37%	0.03%	4.43%
Once a month	2.38%	2.24%	1.05%	1.18%	0.00%	1.87%
A few times per year	1.98%	1.81%	0.65%	0.77%	0.00%	1.48%
(Almost) never	15.84%	14.13%	3.92%	4.78%	0.00%	11.32%
Mean	5.34	5.47	6.28	6.20	6.94	5.71
*Bicycle/e-bike*						
(Almost) daily	19.28%	14.29%	**60.54%**	14.75%	9.89%	25.10%
A few times a week	**26.85%**	22.19%	31.46%	22.66%	17.17%	**25.59%**
Once a week	12.71%	11.71%	5.56%	11.84%	10.13%	10.83%
A few times a month	11.28%	11.59%	1.84%	11.59%	11.21%	9.55%
Once a month	4.92%	5.63%	0.30%	5.57%	6.09%	4.33%
A few times per year	6.52%	8.33%	0.15%	8.15%	10.06%	6.10%
(Almost) never	18.43%	**26.26%**	0.16%	**25.43%**	**35.45%**	18.50%
Mean	4.51	3.98	6.49	4.03	3.42	4.65
*Moped/motorcycle*						
(Almost) daily	4.72%	0.95%	0.00%	1.09%	**97.34%**	5.81%
A few times a week	7.36%	2.01%	0.01%	2.26%	2.62%	3.64%
Once a week	5.85%	2.17%	0.04%	2.37%	0.04%	3.05%
A few times a month	6.48%	3.26%	0.16%	3.48%	0.00%	3.74%
Once a month	3.72%	2.54%	0.35%	2.64%	0.00%	2.46%
A few times per year	4.25%	3.94%	1.58%	3.99%	0.00%	3.44%
(Almost) never	**67.62%**	**85.13%**	**97.86%**	**84.16%**	0.00%	**77.85%**
Mean	2.20	1.43	1.03	1.47	6.97	1.85
*Car as the driver*						
(Almost) daily	12.48%	**39.18%**	8.21%	4.68%	0.00%	18.41%
A few times a week	21.25%	38.78%	15.66%	10.18%	0.00%	23.52%
Once a week	6.39%	6.78%	5.28%	3.91%	0.00%	5.81%
A few times a month	12.48%	7.71%	11.55%	9.77%	0.01%	10.14%
Once a month	4.17%	1.50%	4.33%	4.17%	0.04%	3.25%
A few times per year	9.22%	1.92%	10.71%	11.78%	1.45%	7.28%
(Almost) never	**34.00%**	4.13%	**44.27%**	**55.50%**	**98.51%**	**31.59%**
Mean	3.62	5.84	3.03	2.44	1.02	3.95
*Car as the passenger*						
(Almost) daily	3.71%	2.05%	2.18%	2.80%	**56.74%**	4.82%
A few times a week	17.25%	11.34%	11.85%	14.18%	36.89%	14.86%
Once a week	17.60%	13.81%	14.19%	15.80%	5.27%	15.16%
A few times a month	**24.29%**	**22.73%**	**22.97%**	**23.78%**	1.02%	**22.64%**
Once a month	12.00%	13.39%	13.31%	12.82%	0.07%	12.30%
A few times per year	12.64%	16.83%	16.44%	14.73%	0.01%	14.37%
(Almost) never	12.50%	19.85%	19.06%	15.89%	0.00%	15.85%
Mean	3.88	3.36	3.41	3.63	6.49	3.71
*Bus*						
(Almost) daily	5.09%	0.04%	3.27%	19.31%	0.60%	4.43%
A few times a week	13.85%	0.34%	10.09%	**31.27%**	2.75%	10.33%
Once a week	13.44%	0.93%	11.10%	18.06%	4.54%	9.35%
A few times a month	21.01%	4.17%	19.67%	16.79%	12.03%	14.96%
Once a month	13.86%	7.88%	14.71%	6.59%	13.46%	11.52%
A few times per year	**21.73%**	35.34%	**26.13%**	6.14%	**35.81%**	**25.69%**
(Almost) never	11.02%	**51.30%**	15.02%	1.85%	30.82%	23.72%
Mean	3.66	1.69	3.29	5.14	2.34	3.09
*Tram*						
(Almost) daily	2.67%	0.00%	0.00%	33.08%	11.44%	4.63%
A few times a week	11.80%	0.01%	0.08%	**42.82%**	**29.66%**	9.65%
Once a week	12.32%	0.06%	0.33%	13.13%	18.20%	6.59%
A few times a month	**27.83%**	0.86%	3.04%	8.70%	24.15%	12.89%
Once a month	18.38%	3.79%	8.12%	1.69%	9.37%	10.04%
A few times per year	19.63%	27.10%	35.11%	0.53%	5.88%	22.54%
(Almost) never	7.37%	**68.19%**	**53.31%**	0.06%	1.30%	**33.66%**
Mean	3.64	1.38	1.62	5.95	4.87	2.84
*Metro*						
(Almost) daily	1.46%	0.00%	0.00%	34.46%	6.71%	4.13%
A few times a week	8.41%	0.06%	0.05%	**44.67%**	23.86%	8.37%
Once a week	11.00%	0.28%	0.26%	13.16%	19.27%	6.20%
A few times a month	**23.38%**	2.08%	1.98%	6.30%	**25.30%**	11.22%
Once a month	17.71%	5.61%	5.44%	1.08%	11.83%	9.84%
A few times per year	20.99%	23.64%	23.39%	0.29%	8.66%	19.78%
(Almost) never	17.05%	**68.33%**	**68.87%**	0.05%	4.35%	**40.45%**
Mean	3.21	1.43	1.42	6.04	4.45	2.65
*Train*						
(Almost) daily	4.79%	0.01%	6.69%	9.79%	0.00%	4.04%
A few times a week	10.60%	0.06%	13.44%	17.45%	0.00%	8.27%
Once a week	9.02%	0.19%	10.40%	11.97%	0.00%	6.59%
A few times a month	19.01%	1.57%	19.94%	**20.35%**	0.00%	13.39%
Once a month	16.04%	5.13%	15.30%	13.85%	0.00%	11.81%
A few times per year	**28.46%**	35.19%	**24.69%**	19.80%	0.95%	27.85%
(Almost) never	12.09%	**57.86%**	9.54%	6.78%	**99.05%**	**28.05%**
Mean	3.35	1.51	3.64	4.02	1.01	2.84
*Taxi/ride-hailing*						
(Almost) daily	1.62%	0.00%	0.01%	0.91%	0.00%	0.69%
A few times a week	2.04%	0.01%	0.03%	1.28%	0.00%	0.89%
Once a week	3.06%	0.06%	0.10%	2.15%	0.00%	1.38%
A few times a month	6.57%	0.36%	0.58%	5.15%	0.00%	3.15%
Once a month	6.28%	1.04%	1.42%	5.50%	0.00%	3.44%
A few times per year	23.89%	12.04%	13.83%	23.34%	0.24%	17.42%
(Almost) never	**56.53%**	**86.49%**	**84.04%**	**61.67%**	**99.76%**	**73.03%**
Mean	1.88	1.16	1.19	1.70	1.00	1.48
Modality styles	Multimodal	Drive mostly	Active + Train	PT multimodal	Moped/motorcycle multimodal	

Bold indicates the most highly represented category in each category

**Table 5:** Description and comparison of modality styles

	Class 1 (n = 381)	Class 2 (n = 308)	Class 3 (n = 198)	Class 4 (n = 91)	Class 5 (n = 38)	Overall (n = 1016)	F/χ^2^	*p*-value
	Multimodal	Drive mostly	Active + Train	PT multimodal	Moped/motorcycle multimodal			
City							257.79	< 0.001
Utrecht	45.68	51.13	94.97	0.13	0.00	51.18		
Rotterdam	54.32	48.87	5.03	99.87	100.00	48.82		
Socio-demographics								
Age (mean)	34.81	45.37	37.30	34.32	23.97	38.06	41.41	< 0.001
Gender %							70.40	< 0.001
Male	46.46	41.88	26.77	36.46	97.37	42.33		
Non-male	53.54	58.12	73.23	63.54	2.63	57.67		
Working status %							68.72	< 0.001
Full-time	35.17	42.21	35.86	34.07	44.74	37.70		
Part-time	34.65	32.79	32.83	27.47	42.11	33.37		
Student	17.85	2.60	17.17	24.18	13.16	13.49		
No job/retired	12.34	22.40	14.14	14.29	0.00	15.45		
Education level %							78.02	< 0.001
Low education	13.12	17.53	7.58	14.29	21.05	13.78		
Medium education	31.50	35.06	22.22	40.66	78.95	33.37		
High education	55.38	47.40	70.20	45.05	0.00	52.85		
Living with children %							21.95	< 0.001
Yes	19.95	28.57	17.68	24.18	0.00	21.75		
No	80.05	71.43	82.32	75.82	100.00	78.25		
Household income %							97.04	< 0.001
Less than €980	15.75	4.87	17.68	19.78	52.63	14.57		
Between €980 and €1870	25.20	20.78	19.70	29.67	44.74	23.92		
Between €1870 and €2680	22.57	25.32	23.23	20.88	2.63	22.63		
Between €2680 and €3800	18.37	27.27	22.22	12.09	0.00	20.57		
More than €3800	2.62	3.57	6.57	2.20	0.00	3.54		
Don’t know/don’t want to say	15.49	18.18	10.61	15.38	0.00	14.76		
Neighbourhood spatial factors								
Address density (mean)	3841.17	3358.45	3616.12	4633.92	4221.16	3736.39	15.73	< 0.001
Greenery (mean)	44.89	46.19	46.29	39.25	36.63	44.74	16.04	< 0.001
Travel contexts								
Holding driver’s licence %							223.92	< 0.001
Yes	64.57	95.45	72.73	43.96	0.00	71.24		
No	35.43	4.55	27.27	56.04	100.00	28.76		
Car ownership %							312.90	< 0.001
Personal car	30.71	79.55	20.71	18.68	0.00	41.33		
Household car	14.44	7.47	10.61	7.69	0.00	10.43		
No car	54.86	12.99	68.69	73.63	100.00	48.24		
Bicycle/e-bike ownership %							66.05	< 0.001
Yes	80.58	75.00	100.00	68.13	86.84	81.79		
No	19.42	25.00	0.00	31.87	13.16	18.21		
Public transport subscription %							79.15	< 0.001
Yes	39.11	21.75	60.61	42.86	31.58	38.09		
No	60.89	78.25	39.39	57.14	68.42	61.91		
Car-sharing membership %							23.69	< 0.001
Yes	4.72	1.62	10.10	2.20	0.00	4.43		
No	95.28	98.38	89.90	97.80	100.00	95.57		
(E-)Bike-sharing membership %							63.14	< 0.001
Yes	8.14	1.30	20.71	12.09	0.00	8.56		
No	91.86	98.70	79.29	87.91	100.00	91.44		
Planning trips by smartphone %							107.37	< 0.001
Yes	53.28	29.55	64.65	59.34	97.37	50.50		
No	46.72	70.45	35.35	40.66	2.63	49.50		
Feeling the ease of driving %							86.74	< 0.001
Yes	35.51	79.03	31.08	13.47	0.00	44.29		
No	64.49	20.97	68.92	86.53	100.00	55.71		
Perceiving good PT quality %							36.17	< 0.001
Yes	52.99	50.70	64.90	47.44	2.72	52.26		
No	47.01	49.30	35.10	52.56	97.28	47.74		
Walking way density (mean)	9.88	10.53	11.57	8.60	10.32	10.31	8.18	< 0.001
Cycling way density (mean)	6.14	6.09	7.34	5.78	6.49	6.34	15.91	< 0.001
Bus frequency density (mean)	96.82	83.05	112.40	106.66	80.77	95.96	10.91	< 0.001
Tram/metro frequency density (mean)	103.96	80.79	17.82	179.28	450.73	99.93	48.37	< 0.001
Distance to the nearest main road access ramp (mean)	2.21	2.14	1.97	2.85	2.54	2.21	19.69	< 0.001
Distance to the nearest interchange train station (mean)	4.35	5.31	3.74	4.67	4.69	4.56	15.70	< 0.001
Distance to the nearest train station (mean)	2.09	2.47	1.68	2.28	2.35	2.15	9.72	< 0.001

F = one-way ANOVA tests on continuous characteristics; χ^2^ = chi-square tests on categorical characteristics

#### Multimodal

The *Multimodal* group use most transport modes with moderate frequencies. Their average frequency of using each mode is roughly consistent with the overall sample, but they use taxi/ride-hailing more than other groups. This group of people are equally distributed in the two cities. The *Multimodal* group is relatively young (34.81), highly educated (55.38%), and overrepresented people who share car(s) with family members (14.44%).

#### Drive mostly

The *Drive mostly* group mostly drive cars daily or a few times a week, and they use other modes except walking less frequently. This group of people are equally distributed in the two cities. The average age of the *Drive mostly* group is much older than other groups (45.37), and the proportion of people who are unemployed or retired is also the highest (22.40%). Most people in this group hold a driver’s licence (95.45%) and have a personal car (79.55%), while very few have public transport subscriptions (21.75% versus 38.09% overall sample). Most of them feel comfortable driving (79.03%). The average distances from their residential neighbourhoods to the nearest interchange train station and the nearest train station are the farthest among all modality styles.

#### *Active* + *Train*

The *Active* + *Train* group mostly walk or cycle daily, and most of them take trains at least once a month, higher than the average of the overall sample. Most people in this group are from Utrecht. The *Active* + *Train* group has the highest proportion of non-males (73.23%), highly educated people (70.20%), and high-income people (6.57% with a household income higher than 3800 euros per month). A large proportion of them have no access to a car (68.69%), while all of them have a personal bicycle/e-bike. This group also has the highest proportion of people having public transport subscriptions (60.61%), car-sharing memberships (10.10%), and (e-)bike-sharing memberships (20.71%). The *Active* + *Train* group has the highest proportion of people who think the quality of public transport is good (64.90%). Their residential neighbourhoods have the highest average walking way density, average cycling way density and average bus frequency density, but the lowest average tram/metro frequency density. The average distances from their residential neighbourhoods to the nearest main road access ramp, the nearest interchange train station, and the nearest train station are all the shortest among the five modality styles.

#### PT multimodal

The *PT multimodal* group mostly use buses, trams, metros and trains at moderate to high frequencies. Almost all people in this group are from Rotterdam. This group is relatively young (34.32) and mostly non-male (63.54%). The proportions of students (24.18%), and people with low household income (49.45% lower than 1870 euros per month) are higher than the overall sample. More than half of this group do not hold a driver’s licence (56.04%), most of them have no access to a car (73.63%), and only a small proportion of them feel the ease of driving (13.47%). The residential neighbourhoods of the *PT multimodal* group have the lowest average walking way density and cycling way density, but the average bus frequency density and tram/metro frequency density are both higher than the overall sample.

#### Moped/motorcycle multimodal

The *Moped/motorcycle multimodal* group mostly use moped/motorcycle and car as a passenger daily, and they use tram and metro more frequently than the overall sample. All people assigned to this group are from Rotterdam. This group is much younger (23.97) than other groups, and almost all of them are male (97.37%). Most of this group have full-time (44.74%) or part-time (42.11%) jobs, with low (21.05%) to medium (78.95%) education levels and low household income (97.37% lower than 1870 euros per month). None of them live with children. All of them do not hold a driver’s licence and have no access to a car. None of them are members of car-sharing or (e-)bike-sharing, but most of them use smartphones for trip planning (97.37%). No one in this group feels comfortable driving (also due to the inability to drive), and few of them think the quality of public transport is good (2.72%). Their residential neighbourhoods have much higher tram/metro frequency density than all the other groups.

### Associations between travel contexts and modality styles

To examine the nuances of the associations between travel contexts and modality styles in more detail, we compared all modality styles with each other by performing four weighted multinomial logit regression models with different modality styles as reference classes. All results of the regression models are presented in Table 6, but for brevity, we use Table 7 to summarise the estimated associations between travel contexts and modality styles and describe the results based on it. Table 7 presents that, given a travel contextual factor (in the first column), the probability of belonging to each modality style (in the first row) is higher compared to which modality styles (in the corresponding cell, represented by the abbreviation). For example, if an individual holds a driver’s license, then their probability of belonging to the *Multimodal* class (M) is higher than the *Moped/motorcycle multimodal* class (MM). The results show that travel contextual factors including *licence ownership*, *vehicle ownership*, *mobility service usage*, *subjective attributes*, and *neighbourhood accessibility to transport options* are all associated with individuals’ modality styles.

**Table 6:** Multinomial logistic regression estimating associations between covariates and class membership

	**Ref: Multimodal**				**Ref: Drive mostly**			**Ref: Active + Train**		**Ref: PT multimodal**
	Drive mostly	Active + Train	PT multimodal	Moped/ motorcycle multimodal	Active + Train	PT multimodal	Moped/ motorcycle multimodal	PT multimodal	Moped/ motorcycle multimodal	Moped/ motorcycle multimodal
Intercept	0.388	− 8.060^***^	− 7.031^***^	− 13.781^**^	− 8.448^***^	− 7.420^***^	− 14.169^**^	1.028	− 5.722	− 6.750
Rotterdam (reference: Utrecht)	− 2.156^***^	− 4.416^***^	5.968^***^	8.307^*^	− 2.260^***^	8.123^***^	10.462^*^	10.384^***^	12.723^**^	2.339
Socio-demographics										
Age	0.305	0.805^***^	0.072	− 0.086	0.501^*^	− 0.233	− 0.391	− 0.733^*^	− 0.891	− 0.158
Non-male (reference: male)	0.394	1.031^***^	0.041	− 2.949^*^	0.637	− 0.353	− 3.343^**^	− 0.990	− 3.980^**^	− 2.990^*^
Working status (reference: full-time)										
Part-time	− 0.215	− 0.453	− 0.206	− 3.465	− 0.238	0.010	− 3.250	0.248	− 3.012	− 3.260
Student	− 1.580^*^	0.093	1.017	− 1.794	1.673^*^	2.597^**^	− 0.214	0.924	− 1.887	− 2.811
No job/retired	0.705	− 0.309	− 0.934	− 4.659	− 1.014^*^	− 1.639	− 5.364	− 0.625	− 4.350	− 3.725
Education level (reference: low education)										
Medium education	0.369	− 0.651	0.410	− 0.492	− 1.020	0.041	− 0.861	1.061	0.159	− 0.902
High education	− 0.140	− 0.285	0.597	− 2.376	− 0.145	0.737	− 2.236	0.882	− 2.092	− 2.973
Living with children	− 0.038	0.334	0.797	− 1.773	0.371	0.835	− 1.735	0.463	− 2.106	− 2.570
Household income (reference: less than €980)										
Between €980 and €1870	0.223	− 1.425^*^	0.320	− 2.052	− 1.648	0.097	− 2.275	1.745	− 0.627	− 2.372
Between €1870 and €2680	0.110	− 1.053	− 0.483	0.264	− 1.163	− 0.592	0.154	0.570	1.317	0.747
Between €2680 and €3800	0.342	− 0.366	− 0.280	− 5.924	− 0.708	− 0.622	− 6.266	0.086	− 5.558	− 5.644
More than €3800	0.578	1.104	0.181	− 14.866^**^	0.526	− 0.398	− 15.444^**^	− 0.924	− 15.970^**^	− 15.046^***^
Neighbourhood spatial factors										
Address density	0.531	0.633	0.965	− 2.579^*^	0.102	0.434	− 3.110^**^	0.332	− 3.212^**^	− 3.544^**^
Greenery	0.149	0.201	0.346	− 1.750^*^	0.052	0.198	− 1.899^*^	0.146	− 1.951^*^	− 2.096^*^
Travel contexts										
Holding driver’s licence	1.658^*^	1.294^**^	− 0.550	− 5.344^*^	− 0.364	− 2.208^*^	− 7.002^**^	− 1.844^*^	− 6.638^**^	− 4.794^*^
Car ownership (reference: personal car)										
Household car	− 1.007^*^	− 0.261	− 0.966	− 2.047	0.746	0.041	− 1.040	− 0.705	− 1.786	− 1.081
No car	− 1.388^***^	1.259^**^	0.561	4.857	2.647^***^	1.948^**^	6.245^*^	− 0.698	3.598	4.297
Having a personal bicycle/e-bike	− 0.762^*^	7.039^***^	− 1.025^*^	− 1.925^*^	7.801^***^	− 0.263	− 1.163	− 8.064^***^	− 8.964^***^	− 0.900
Having public transport subscription	− 0.985^**^	0.736^*^	0.397	0.415	1.721^***^	1.381^**^	1.400	− 0.339	− 0.321	0.018
Car-sharing member	− 1.321	− 0.880	− 2.322	− 0.127	0.441	− 1.001	1.194	− 1.442	0.753	2.195
(E-)Bike-sharing member	− 1.332	0.955	0.786	− 0.858	2.287^**^	2.119	0.474	− 0.168	− 1.813	− 1.645
Planning trips by smartphone	− 0.918^**^	0.230	0.506	3.634^**^	1.149^**^	1.424^**^	4.552^***^	0.275	3.403^**^	3.128^**^
Feeling the ease of driving	1.304^***^	− 0.189	− 1.346	− 1.967	− 1.493^**^	− 2.650^**^	− 3.271	− 1.157	− 1.778	− 0.621
Perceiving good PT quality	− 0.289	0.165	− 0.362	− 3.334	0.455	− 0.073	− 3.045	− 0.528	− 3.500	− 2.972
Walking way density	0.176	0.090	− 0.277	0.827	− 0.087	− 0.453	0.651	− 0.366	0.738	1.104^*^
Cycling way density	0.049	0.343^*^	− 0.083	− 3.319^*^	0.294	− 0.131	− 3.368^*^	− 0.425	− 3.661^**^	− 3.236^*^
Bus frequency density	− 0.251	− 0.364	0.028	− 0.742	− 0.113	0.278	− 0.491	0.391	− 0.379	− 0.770
Tram/metro (transit) frequency density	0.012	0.128	0.146	2.433^*^	0.116	0.133	2.421^*^	0.018	2.305^*^	2.288^*^
Distance to the nearest main road access ramp	0.201	− 0.183	0.448	1.122	− 0.385	0.246	0.920	0.631	1.305	0.674
Distance to the nearest interchange train station	0.607^*^	0.333	1.291	− 0.057	− 0.274	0.684	− 0.663	0.958	− 0.390	− 1.348
Distance to the nearest train station	0.088	− 0.423	− 0.602	0.488	− 0.510	− 0.689	0.401	− 0.179	0.911	1.090^*^

*p <.05; ^**^p <.01; ^***^p <.001

**Table 7:** A summary of associations between travel contexts and modality styles

**Travel contexts**	Multimodal (M) Higher than	Drive mostly (D) Higher than	Active + Train (AT) Higher than	PT multimodal (PT) Higher than	Moped/ motorcycle multimodal (MM) Higher than
Holding driver’s licence	MM	M, PT, MM	M, PT, MM	MM	
Personal car	AT	M, AT, PT, MM			
Household car	D				
No car	D		M, D	D	D
Having a personal bicycle/e-bike	D, PT, MM		M, D, PT, MM		
Having public transport subscription	D		M, D	D	
Car-sharing member					
(E-)Bike-sharing member			D		
Planning trips by smartphone	D		D	D	M, D, AT, PT
Feeling the ease of driving		M, AT, PT			
Perceiving good PT quality					
Walking way density					PT
Cycling way density	MM	MM	M, MM	MM	
Bus frequency density					
Tram/metro frequency density					M, D, AT, PT
Distance to the nearest main road access ramp					
Distance to the nearest interchange train station		M			
Distance to the nearest train station					PT

Given the travel contextual factors (in the first column), the probability of belonging to each modality style (in the first row) is higher compared to which modality styles (in the corresponding cell, represented by the abbreviation)

#### Licence and vehicle ownership

Individuals who hold a driver’s licence are more likely to fall into the *Drive mostly* class and the *Active* + *Train* class than the other three classes and are least likely to be *Moped/motorcycle multimodal*. Individuals having a personal car have a higher probability of being in the *Drive mostly* class than all the other classes and are more likely to belong to the *Multimodal* class than the *Active* + *Train* class. Those who have a personal bicycle/e-bike are more likely to belong to the *Active* + *Train* class than all the other classes and are more likely to belong to the *Multimodal* class among the remaining classes.

#### Mobility service usage

Individuals who have a public transport subscription are more likely to belong to the *Active* + *Train* class than the *Multimodal* and *Drive mostly* classes, and less likely to be in *Drive mostly* than *Multimodal* and *PT multimodal*. Those who are (e-)bike-sharing members are more likely to belong to the *Active* + *Train* class than the *Drive mostly* class. Individuals using smartphones for trip planning are more likely to belong to the *Moped/motorcycle multimodal* class than all the other classes and are less likely to belong to the *Drive mostly* class than all the other classes.

#### Subjective attributes (perceptions of travel options)

Individuals who feel comfortable driving are more likely to belong to the *Drive mostly* class than the *Multimodal*, *Active* + *Train* and *PT multimodal* classes.

#### Neighbourhood accessibility to transport options

Individuals living in neighbourhoods with higher walking way density are more likely to fall into the *Moped/motorcycle multimodal* class than the *PT multimodal* class. Individuals living in neighbourhoods with higher cycling way density are more likely to fall into the *Active* + *Train* class than the *Multimodal* and *Moped/motorcycle multimodal* classes, and less likely to belong to the *Moped/motorcycle multimodal* class than all the other classes. For individuals living in neighbourhoods with higher tram/metro frequency density, their probability of falling into the *Moped/motorcycle multimodal* class is higher than all the other classes. Individuals living farther from the nearest interchange train station are more likely to belong to the *Drive mostly* class than the *Multimodal* class, and those who live farther from the nearest train station are more likely to belong to the *Moped/motorcycle multimodal* class compared to the *PT multimodal* class.

## Discussion

### Discussion on the identification of modality styles

Five modality styles were identified in this study: (1) *Multimodal*, (2) *Drive mostly*, (3) *Active* + *Train*, (4) *PT multimodal*, and (5) *Moped/motorcycle multimodal*. *Drive mostly* is the only monomodal pattern, and the other four modality styles are multimodal. Previous studies found similar modality styles to *Drive mostly* and *PT multimodal* (Lee et al. 2020; Molin et al. 2016; Ton et al. 2020). However, they often identified more than one car-dominated modality styles without distinguishing between car drivers and car passengers (Faber et al. 2022; Schneider et al. 2021). In contrast, our study only identified one car-dominated modality style, which mainly uses car as a driver. Considering the distinction between car drivers and car passengers, we also identified a modality style that mainly uses car as a passenger and uses several other transport modes at equally high frequencies (*Moped/motorcycle multimodal*). The results suggest that car use varies in the role of driver and passenger, with drivers being more car-dependent and monomodal, and passengers being more multimodal. Nevertheless, this difference may also be because our study is based on highly urbanised areas in the Netherlands, which are less car-oriented (Nello-Deakin and Brömmelstroet 2021). *PT multimodal* identified in this study differs from previous studies, with not only higher than average frequencies of using bus, tram, metro and train, but also higher than average frequency of using taxi/ride-hailing. Since most other groups never use taxi/ride-hailing, this suggests that taxi/ride-hailing might serve as a complement to public transport.

Three modality styles identified in this study *Multimodal*, *Active* + *Train*, and *Moped/motorcycle multimodal* are different from modality styles that have been found in previous studies in terms of the combination of modes and their relative share. The *Multimodal* group uses most of the transport modes, with no mode dominating, whereas modality styles found in previous studies generally had certain dominant modes (De Haas et al. 2018; Krueger et al. 2018). This is presumably because car and public transport, treated as single modes in previous studies, were split into several distinct modes in this study, reducing their dominance. The *Multimodal* group also uses taxi/ride-hailing more frequently than other groups. As studies found that multimodal travellers are familiar with different transport modes and are more flexible in their mode choice (Buehler and Hamre 2015; Vij et al. 2011), taxi/ride-hailing, an uncommon transport mode in the Netherlands could also be an alternative travel option for the *Multimodal* group. Previous studies have also found modality styles with a combination of active modes and public transport (Janke et al. 2021; Ton et al. 2020). Accounting for distinct modes of public transport, we further found that active modes are more likely to be used in conjunction with train rather than local public transport (bus, tram, metro), which may also be due to the presence of OV-fiets, an integrated system of train and bike-sharing operated by NS (Nederlandse Spoorwegen, the main passenger railway operator in the Netherlands) (Villwock-Witte and van Grol 2015). Bus, tram and metro were also found to be used differently, with bus being used more frequently in the *Active* + *Train* class, while tram and metro being used more frequently in the *Moped/motorcycle multimodal* class. This suggests that different forms of local public transport may serve different traveller segments. *Moped/motorcycle multimodal* is a unique modality style that mainly uses moped/motorcycle, tram, metro and car as a passenger, while previous research often lacked attention to these constitute modes. Although only a small proportion of people were found to be in this class, their high frequency of using moped/motorcycle warrants attention because moped/motorcycle is the biggest source of noise nuisance in the Netherlands^10^ and the most dangerous mode of road travel in many areas of the world^11^.

By considering more transport options and distinguishing between different forms of public transport and car use, this study uncovered three new forms of multimodality and informed the understanding of the roles of specific transport modes in the modal combination of daily travel (e.g., train, bus, tram, and metro). The findings suggest the necessity to consider a broader and more detailed set of transport options in the identification of present-day multimodal modality styles.

### Discussion on the associations between travel contexts and modality styles

The results show that an individual’s travel context is associated with their probability of falling into a specific modality style (Table 7). Car-related travel contextual factors, including licence ownership, car ownership and feeling the ease of driving are strongly associated with whether an individual is monomodal (as a car driver) or multimodal. Other contextual factors, including bicycle/e-bike ownership, the use of mobility services, and neighbourhood accessibility to transport options are associated with which form of multimodality an individual is more likely to belong to.

Individuals in the *Drive mostly* class were found to typically have a driver’s licence, have a personal car and feel comfortable driving, consistent with previous research (Molin et al. 2016; Ton et al. 2020). We further found that the *Drive mostly* group tend to live farther away from the nearest interchange train stations (central stations). Since both Rotterdam and Utrecht central stations are located in the city centre, these people may live in peripheral areas and need to travel longer distances. Also, since they experience the ease of driving, they may regard driving as superior to other options to meet their travel needs and drive out of choice (Lavery et al. 2013).

Individuals in the *Active* + *Train* class have similar characteristics to the *Drive mostly* class in terms of socio-demographics and neighbourhood spatial factors, while their travel contexts differed in several ways. One of the main differences is that although individuals in the *Active* + *Train* class also mostly have a driver’s licence, they typically do not have a personal/household car. Individuals in the *Active* + *Train* class are more likely to own a personal bicycle/e-bike and live in neighbourhoods with higher cycling way density. They are also more likely to be (e-)bike-sharing members and have public transport subscriptions, which further suggests that they may be users of OV-Fiets. Therefore, despite being able to drive, and having a sufficient income (to purchase a car), they are likely to live car-free by choice and use active modes more (Paijmans and Pojani 2021).

Individuals in the *Multimodal* class share similarities in travel contexts with those in the *Active* + *Train* class, but individuals in the *Multimodal* class were found more likely to own either a personal car or a household car. Having a personal bicycle/e-bike, subscribing to public transport and living in neighbourhoods with higher cycling way density are associated with a higher probability of belonging to the *Multimodal* class compared to several other classes. The findings indicate that individuals in the *Multimodal* class have better access to various transport options and a better ability to use or afford the mobility services of their choice. Thus, they are likely to deliberately choose the optimal travel mode for every given trip based on a sufficient set of transport options and being multimodal out of choice (Kroesen 2014; Kuhnimhof et al. 2006).

Individuals in the *PT multimodal* class are found more likely to subscribe to public transport and they are also more likely to be students. Therefore, this group of people may benefit from student travel products provided by the national railway and local transit companies, allowing them to travel by public transport for free or with a discount. According to the description (Table 5), a lower proportion of individuals in the *PT multimodal* group perceive PT as being of good quality, which may indicate that they use such a combination of transport modes by necessity rather than willingness, so-called captive transit users (Jacques et al. 2013).

Individuals in the *Moped/motorcycle multimodal* class live in neighbourhoods with lower cycling way density and higher tram/metro frequency density, which may lead them to frequently use tram/metro but hardly cycle. Individuals in this group typically do not have a driver’s licence or a car, and since they use mopeds/motorcycles and use cars as passengers on a daily basis, they may prefer private motorised modes. However, the inability to drive and purchase a car may constrain them from driving and force them to use suboptimal alternatives. They are more likely to use smartphones for trip planning compared to other groups, but most of them do not have public transport subscriptions according to the description in Table 5. Therefore, they may be more inclined to use digital mobility products instead of traditional travel products. We also found that although this group of people frequently use tram/metro, they mostly do not feel the quality of public transport is good and do not have public transport subscriptions according to the description in Table 5. This group of people are thus likely to be captive-transit users and since they also have the tendency to use private vehicles, their travel needs deserve attention from transport authorities.

According to the associations between travel contexts and modality styles, we can formulate the following hypotheses about the underlying mechanisms of falling into a specific modality style: **(1) Being monomodal due to habitual behaviour**: the *Drive mostly* group are car-dependent, and they habitually drive despite access to other travel options. (**2) Being multimodal by using a combination of optimal options:** the *Multimodal* and *Active* + *Train* groups both have good accessibility to various transport options and be able to afford or use different mobility services such as PT subscriptions and shared mobility, which enable them to use a combination of optimal options. This suggests Kuhnimhof et al. (2006)’s assumption that multimodality can be a result of situational optimization on one’s daily travel. **(3) Being multimodal due to constraints on using certain modes**: the *PT multimodal* and *Moped/motorcycle multimodal* groups live in neighbourhoods with worse walking or cycling environments and have no driver’s licence or car, the constraints on accessibility and affordability lead them to use a combination of sub-optimal options for fulling their needs. This suggests that travellers can also be captive to multimodality because of the inability to use the preferred mode, especially for the *Moped/motorcycle multimodal* group.

The above discussion may provide evidence for the debate about whether multimodality is by choice or necessity and suggest that both mechanisms probably exist but underlie different forms of multimodality. Previous studies have distinguished users of a certain transport mode as choice users, captive users and captive-by-choice users based on their motivations (Jacques et al. 2013; Van Lierop and El-Geneidy 2017). Our research findings further suggest that such classification may be extended to the use of multiple transport modes and contribute to the understanding of traveller segments.

### Implications for policy and practice

Our research findings may inform transport policy and planning that aims to encourage sustainable forms of multimodality and reduce car dependence. Tailoring the interventions to different traveller segments may be key to policy effectiveness (Dacko and Spalteholz 2014), so we propose the following recommendations on optimizing travel context for each modality style.

The *Drive mostly* class is the least sustainable among all classes. This group of people are characterized as feeling comfortable driving, and thus setting stricter driving regulations for example lower speed limits in certain areas and fewer parking spaces in city centres may make people re-examine driving and take more alternatives into account (Timmer et al. 2023a, b). Moreover, although we found the *Drive mostly* group have higher incomes, they rarely own and use mobility services and products, so they may have little willingness to use transport modes and services other than personal cars (Sharmeen and Timmermans 2014). Also, because they live in areas farther from the city centre, driving may be a necessity for them. Therefore, encouraging them to replace traditional fuel vehicles with electric vehicles may be a more effective way to promote their sustainable travel.

The *Active* + *Train* class is the most sustainable among all classes, so their travel contexts should support them to retain this modality style. As we found this group of people are more likely to be (e-)bike-sharing members and subscribe to public transport, offering tailor-made mobility products such as bundles of shared mobility and public transport (Montes et al. 2023). We found that most people in the *Active* + *Train* group live in Utrecht, a city known for its excellent cycling environment. Also, we found the neighbourhoods where the *Active* + *Train* group live have more cycling facilities than where other groups live. Therefore, even in a city that is already very bike-friendly, planning more bike lanes and designing a well-connected urban bike system has the potential to maintain and attract travellers to adopt such a sustainable travel pattern. Furthermore, the deployment of shared (e-)bikes could follow the distribution of cycling facilities, where there are likely to be more potential users.

The *Multimodal* class is less sustainable than the *Active* + *Train* due to the greater use of cars. Individuals in this group are younger than *Active* + *Train* and *Drive mostly*, and because their travel contexts have certain similarities, the *Multimodal* group could potentially switch to one of the two modality styles when get older. To lead them to *Active* + *Train* and prevent them from being *Drive mostly*, it is important that they have better experiences using public transport and active modes than driving. Thus, except for the same measures to be taken as the *Drive mostly* and *Active* + *Train* classes, improving their experiences with public transport using measures related to reliability and comfort should be prioritized (Van Lierop et al. 2018).

The *PT multimodal* class is the second most sustainable, and travellers of this class have the potential to switch to *Active* + *Train.* Travellers of the *PT multimodal* class are more likely to be students and benefit from student travel products, so they use a combination of local public transport and train. When they leave education, they may change their way of travelling due to changes in travel needs. Therefore, policies should consider how to keep them using different public transport modes or lead them to *Active* + *Train*, depending on their new travel needs. Providing the group of people who start their career by tailoring public transport products, for example, allowing them to switch the student public transport product directly to another season ticket may be a way to retain them to be *PT multimodal*. For this group of people, the need to travel longer distances may make them use public transport more than active modes, so a (shared) e-bike may be better meet their travel needs as it is faster than a traditional bicycle and enables travelling longer distances (De Haas et al. 2022). Thus, subsidizing the purchase of e-bikes, especially for those who are about to graduate and enter the job market may be an important solution to enable them to travel more easily and sustainably.

The *Moped/motorcycle multimodal* class is a less sustainable class using a combination of motorised modes, but travellers of this class were also found may experience disadvantages in daily travel. Poorer cycling facilities in their neighbourhoods may make them travel more by mopeds/motorcycles instead of cycling, so it is important to improve cycling facilities around their residential neighbourhoods, typically those neighbourhoods with lower density and greenery but close to the tram/metro stations. Increasing the connectivity between tram/metro stations and bike lanes is essentially important since we found although their neighbourhoods have the highest tram/metro frequency density, the cycling way density is the lowest. Their frequent use of mopeds/motorcycles can cause heavy noise nuisance. Encouraging them to use an electric alternative to the moped/motorcycle, such as an e-bike or e-moped, could also be a solution to make them travel more sustainably. As we also found they are more likely to use digital devices and services, offering them tailor-made digital mobility services such as bundling of public transport and shared e-bike/e-moped may also enable them to travel more by transport modes producing less air and noise pollution.

### Limitations and future research

Future research may extend the present study in the following ways. First, the way in which frequency of mode use was asked in the survey may lead to varied interpretations and potential biases in identifying and interpreting modality styles. Respondents were asked how many days they use a certain mode of travel in a year, but we did not further explain what “travel” accounts for. This ambiguity may lead to respondents’ different understandings of this question, such as whether connecting/access trips, especially walking trips—a mode often undercounted in travel surveys (Buehler and Pucher 2024), are included. If individuals’ understandings vary randomly, clustering (who are clustered into the same class) may be less biased; however, if linked to personal characteristics or mobility behaviours, clustering can be skewed. Regardless, the ambiguity hinders the accurate interpretation of the identified modality styles, in particular how walking is combined with the use of other modes across traveller groups. Future surveys should clearly define “mode use for travel” to minimise ambiguity.

Second, although our study has extended the set of transport modes used to identify modality styles, more details can be considered in future research. As shared modes, in particular shared micro-mobility has been used more in the daily travel, they may substitute or complement private and public transport modes (Reck et al. 2022), and result in new forms of multimodality. Thus, future research could further distinguish shared modes from private modes to see their roles in multimodality. Also, the role of different transport modes could be better investigated by distinguishing the specific travel purposes, the days of the week (workdays or weekends) and under what weather conditions they are used. These distinguishments could be insightful for understanding the role of different modes in daily travel.

Third, studying how individuals experience different forms of multimodality can offer additional insights into encouraging multimodality. Our study found that different forms of multimodality may be motivated by different reasons, some out of choice and some out of necessity, so individuals may experience them differently. Taking individuals’ experiences into account is therefore important for multimodal transport policies to promote multimodality in a more equitable way.

Forth, longitudinal data and studies focusing on the changes in modality styles and their determinants are needed. Although our study found that an individual’s travel context is associated with which modality style they belong to, the cross-sectional design was unable to tell us whether certain travel contexts result in specific modality styles and how individuals’ modality styles will change when the travel contexts change. Answering this question can add more knowledge for promoting specific forms of multimodality by modifying travel contexts. Since our findings also show that different modality styles correspond to different age groups, travellers potentially switch to other modality styles when they get older. Thus, it will be interesting to investigate the change in modality styles and their relationship with travel contexts over time.

## Conclusion

This study identified five modality styles based on a detailed set of transport modes, including three forms of multimodality different from modality styles that have been found in previous studies in terms of the combination of modes and their relative share. By distinguishing between car drivers and passengers, the only monomodal pattern that predominantly uses car as a driver was found, suggesting that drivers are more car-dependent than passengers. Multimodal travel patterns found in previous studies are often dominated by cars, active modes or public transport, but this study additionally found a multimodal pattern dominated by mopeds/motorcycles. Different modes of public transport were found to appear in different forms of multimodality, suggesting that train, bus, tram and metro may serve different traveller segments. These findings indicate that every single mode could play a distinct role in multimodality and the way in which individuals combine modes for their unique travel needs deserves attention from researchers and policymakers.

The results show that the probability of an individual falling into a specific modality style was associated with their travel contexts. An individual's modality style was found to be largely determined by car ownership, driver's licence ownership and their perception of the ease of driving, suggesting that even in less car-dependent urban contexts, driving is often an option considered superior to others (Buehler and Hamre 2015; Klinger 2017). Moreover, people who use mobility services including public transport subscriptions, (e-)bike-sharing and online trip planners were found to be more likely to present modality styles with combinations of active modes and public transport.

The research findings allow us to better understand the travel behaviour and needs of different traveller segments and offer tailored interventions for each modality style, either maintaining or switching to a more sustainable form of multimodality. We recommended promoting more sustainable forms of multimodality not only through hard policies aimed at reducing the ease of driving but also through soft policies to encourage the purchase and use of e-bikes. Importantly, mobility services such as (e-)bike-sharing should be better integrated into the urban mobility system.

## Data Availability

No datasets were generated or analysed during the current study.

## Appendix

**See **Table 8.

**Table 8:** Variables in the analysis and their data sources

	Variable	Type	Data source
Frequency of mode use	Walking	Ordinal	Survey
	Bicycle/e-bike	Ordinal	Survey
	Moped/motorcycle	Ordinal	Survey
	Car as a driver	Ordinal	Survey
	Car as a passenger	Ordinal	Survey
	Bus	Ordinal	Survey
	Tram	Ordinal	Survey
	Metro	Ordinal	Survey
	Train	Ordinal	Survey
	Taxi/ride-hailing	Ordinal	Survey
Socio-demographics	Age	Continuous	Survey
	Gender	Binary	Survey
	Working status	Categorical	Survey
	Education level	Categorical	Survey
	Living with children	Binary	Survey
	Household income	Categorical	Survey
Neighbourhood spatial factors (Residence PC4)	Address density	Continuous	CBS
	Greenery	Continuous	RIVM
Travel contexts			
Vehicle ownership	Car ownership	Categorical	Survey
	Bicycle/e-bike ownership	Binary	Survey
	Moped/motorcycle ownership	Binary	Survey
Licence ownership	Holding driver’s licence	Binary	Survey
Mobility service usage	Public transport subscription	Binary	Survey
	Car-sharing membership	Binary	Survey
	(E-)Bike-sharing membership	Binary	Survey
	Electric moped-sharing membership	Binary	Survey
	Planning trips by smartphone	Binary	Survey
Subjective attributes	Feeling the ease of driving	Binary	Survey
	Perceiving good public transport quality	Binary	Survey
Neighbourhood accessibility to transport options	Walking way density	Continuous	Open Street Map
	Cycling way density	Continuous	Open Street Map
	Bus frequency density	Continuous	GTFS (OV api)
	Tram/metro frequency density	Continuous	GTFS (OV api)
	Distance to the nearest main road access ramp	Continuous	CBS
	Distance to the nearest interchange train station	Continuous	CBS
	Distance to the nearest train station	Continuous	CBS

According to CBS (Statistics Netherlands), an interchange train station is large railway station with multiple connections to other railway stations, which are classified as ‘large interchange station’ or ‘important interchange station’ on the official railway map published by the Dutch Railways. (https://www.cbs.nl/en-gb/news/2011/07/two-in-every-three-people-in-the-netherlands-live-within-5-kilometres-from-a-railway-station)

## Footnotes

1 https://www.wri.org/insights/us-cities-multi-modal-transportation-benefits

2 https://www.tn.gov/health/cedep/environmental/healthy-places/healthy-places/active-transportation/at/multimodal-transportation.html

3 CBS (statistics Netherlands) (2021). Onderweg in Nederland (ODiN) 2021—Onderzoeksbeschrijving. https://www.cbs.nl/nl-nl/longread/rapportages/2022/onderweg-in-nederland--odin---2021-onderzoeksbeschrijving

4 KiM (Netherlands Institute for Transport Policy Analysis) (2022). Bijlage bij Kerncijfers Mobiliteit 2022. https://www.kimnet.nl/mobiliteitsbeeld/publicaties/publicaties/2022/11/15/kerncijfers-mobiliteit-2022

5 KiM (Netherlands Institute for Transport Policy Analysis) (2022). Bijlage bij Kerncijfers Mobiliteit 2022. https://www.kimnet.nl/mobiliteitsbeeld/publicaties/publicaties/2022/11/15/kerncijfers-mobiliteit-2022

6 CBS (Statistics Netherlands): https://www.cbs.nl/nl-nl/onze-diensten/methoden/begrippen/omgevingsadressendichtheid-van-een-adres

7 RIVM (National Institute for Public Health and the Environment): https://www.atlasleefomgeving.nl/kaarten

8 GTFS (General Transit Feed Specification): https://gtfs.org/

9 CBS (Statistics Netherlands): https://opendata.cbs.nl/statline/portal.html?_la=nl%26_catalog=CBS%26tableId=84953NED%26_theme=413

10 https://rivm.openrepository.com/handle/10029/623746 (Van Poll, R., Breugelmans, O., Houthuijs, D., & van Kamp, I. (2020). Perception of Living Environment in the Netherlands: Disturbances Survey 2016.).

11 https://road-safety.transport.ec.europa.eu/eu-road-safety-policy/priorities/safe-vehicles/archive/motorcycles_en
